# To Reduce Gold and Silver Scraps and Filings to Plate

**Published:** 1871-03

**Authors:** T. L. Buckingham


### ARTICLE VI.

### *To Reduce Gold and Silver Scraps and Filings to Plate.*

By T. L. Buckingham, D. D. S.

Now, when rubber work has nearly run its course, as I hope it has, and patients are beginning to demand better materials, it may not be out of the way to say a few words in regard to reducing gold and silver scraps and filings to plate, so that they can be worked up. This information is required more by the younger members of our profession than by those older, as many of them have come into the profession since rubber-work was introduced, and have seen little or no metal work done; in fact, we have occasionally students who have been in practice four or five years, and have never done a piece of metal work. I think the profession at large will agree with me in saying, that continuous gum, gold, or even silver work, is much better than this cheap rubber work, that is so generally used.

It does one good to see, as we do occasionally, a piece of gold work that has been worn ten or fifteen years, and looks nearly as well as it did when made. There is about the same comparison between it and this cheap rubber work that there is between a good gold one and an amalgam filling.

As we cannot afford to throw away the gold and silver scraps and filings as we do those of rubber, it is well to know some process to reduce them to plate. The process I have adopted, and, if tried by others, I think will be found very good, I will not say the best, for the same results may be arrived at by other processes, is to first keep the scraps and filings as clean as possible, for, with the greatest care, a large amount of foreign matter will get in. We have constantly to use zinc and lead for our casts, tin sometimes for patterns, and the wearing of the files adds iron or steel; these, with the plaster and other substances that will get in, make quite a conglomerate mass. The first thing I do with my gold scraps and filings is to pick out all the large pieces of gold with a pair of pliers, removing all the large pieces of the other baser metal. Then sieve the small scraps and filings through a sieve, which I have made out of a tin blacking box lid by punching it full of holes with a plate punch as far in as I could reach, and then making holes in the centre with a small punch and hammer. It may be possible to buy small sieves, but I have not seen them for sale at any of the depots. This separates the filings and very small pieces from the scraps. The scraps are now spread out on a piece of clean paper, and all the pieces of base metals are picked out with a pair of pliers. Through the filings is first passed a magnet, which removes all the iron and steel; they are then put into an evaporating dish or Florence flask, and some dilute nitric acid poured on them. This dissolves the silver and baser metals, except the tin, which is oxidized, and remains with the gold fillings. When the acid ceases acting it is poured off and kept, to obtain the silver which it contains. The filings should be washed several times with fresh water and then dried, when they are ready for melting. The larger pieces of scraps and parts of old plates that contain no solder, and are firm enough to make plates out of again, should be melted together, and when in a melted state a few pieces of saltpetre (nitrate of potassa) should be thrown into the crucible. Nitrate of potassa is decomposed, the oxygen oxidizes the baser metals that are present, and they are absorbed by the crucible, but it is exceedingly difficult to get rid of all the tin in this way; it is one of the hardest metals to separate from gold that we have, and there will be nearly always a small portion remaining, but as it adds to the elasticity and hardness of the gold the small quantity that is left does little harm. We cannot be certain of the quality of gold refined in this way—it may be more or less than eighteen karat fine; the platina and tin changes the color very much, and also gives it hardness and elasticity, so that this gold should be used for making clasps and stays for the teeth; for which purpose it answers better than the gold plates alloyed from the coin.

To reduce silver scraps and filings, we proceed in the same manner that we do with gold, separating the larger pieces that will do to melt without being refined and melting them with a little borax, or if they contain solder, and it is necessary to refine them, throwing into the crucible a little saltpetre until they are melted. By using a sufficient quantity of saltpetre they may be made nearly pure silver. The scraps which contain solder and the filings, after being sieved and having a magnet passed through them to remove the iron, should be put into an evaporating dish or Florence flask, and have dilute nitric acid poured on them; this, with a gentle heat, will dissolve the silver. The acid should be added until it ceases acting, which may be known by no more red fumes coming over when fresh acid is added. This operation should be performed in a chimney place, or where there is a draft of air to carry the fumes out of the laboratory, as they are poisonous to inhale, and oxidize the instruments when they come in contact with them. The residue that is left in the flask, after the acid containing the silver is poured off, usually has enough of gold filings with it to pay for the trouble of refining the silver. It is singular with what tenacity gold will stick to nearly everything it comes in contact with, except a man's pocket book, and there it almost appears to undergo a spontaneous evaporation.

The next part of the process is to obtain the silver from the solution. This may be done in several ways. If a piece of copper plate be suspended in the fluid that contains the silver, the nitric acid will leave the silver and combine with the copper. The silver will fall down in a dark, grayish, crystalline mass, and only requires to be dried and melted with a little borax, or the solution may be diluted with twice its bulk of water or more, and a solution of common salt (chloride of sodium) poured into it, taking care not to pour too much in at one time. A white curdy precipitate forms, which soon falls to the bottom of the vessel. When no more of this precipitate is formed when fresh additions of the solution salt is added, it may be allowed to settle, and the fluid poured off and thrown away, as it contains nothing worth saving. The chloride of silver should now be washed by pouring over it some fresh water, and stirring it up; when it has settled, pour the water off; by repeating this operation several times, the chloride may be washed perfectly clean.

The next part of the operation is to reduce the chloride of silver. The chloride should be covered with fresh water, and add a small portion of sulphuric acid; then put a few small pieces of zinc in the vessel, and let it stand for a day. The chlorine will leave the silver and combine with the zinc. When all the chloride has been reduced, it is better to use an excess of zinc and sulphuric acid, to ascertain that the chloride is all reduced. The zinc, if any remains, should be removed, and fresh sulphuric acid added, so as to dissolve any zinc that might remain. As much of the fluid is now to be poured off as can be, without losing the silver, fresh water poured on, and this is to be repeated until the water comes off tasteless. The silver will be in a dark grayish powder, much finer than when it is precipitated with copper, and only requires to be melted and rolled to be brought into plates. There are other processes of reducing the chloride of silver, but the above are the simplest and least expensive.

Pure silver is always useful in the labratory. We use it for making solder; two parts silver and one of fine brass makes the best silver solder. The silver should be melted first, with a little borax, and, while melted, the brass dropped in. If they are both melted together a portion of the brass will be burnt out, and the solder will not be so good.

Gold alloyed to the required karat with silver solder makes the best gold solder. Pure silver is too soft to make plates, but it can be used for rimming above the teeth, as it can be bent more easily than coin. Silver alloyed with platina makes a stiff plate, and is preferable to coin as it contains no copper.

Silver wire dipped in nitric acid forms nitrate of silver on the surface of the wire, and the caustic may be applied in this way to the gums, or to the pulp of a tooth, or into an alveolar abscess, more easily than it can from a solid stick of caustic.—*The Dental Times.*

